# *The Organon* and Materia Medica Club of the Bay Cities of California

**Published:** 1896-04

**Authors:** W. E. Ledyard

**Affiliations:** Secretary


W. E. LEDYARD, *Secretary.*

Reported by Eleanor F. Martin, M. D.

---

### *THE ORGANON* AND MATERIA MEDICA CLUB OF THE BAY CITIES OF CALIFORNIA.

The regular semi-monthly meeting was held at the office of Dr. George H. Martin, 606 Sutter Street, San Francisco, Friday evening, November 1st, 1895.

Members present: Drs. J. M. Selfridge, George H. Martin,

A. McNeil, S. E. Chapman, M. T. Wilson, W. E. Ledyard, and M. F. Underwood. Visitor, Mr. A. Johnson.

The meeting was called to order at 8.15 by the President, Dr. J. M. Selfridge. The minutes of the previous meeting were read by the Secretary, and after a slight correction by Dr. Chapman were approved.

Dr. Wilson stated that the case of the young man with bowel trouble related by him at the last meeting was now apparently well.

Dr. McNeil then said—I have a question on medicine that I would like to have discussed by the members of this Club. I would like to hear something upon *appendicitis.* I want to know if it is a fact that every time a man has a pain in his bowels you must call it appendicitis, and operate upon him to find grape-seeds.

Dr. Selfridge—There have been many cases in which no grape-seeds were found. I believe it comes in connection with chronic constipation. Nearly every attack of appendicitis is preceded by constipation. I cannot say whether it is the presence of a foreign body in the appendix which causes it or not. It is simply the catarrhal form if it gets well without an operation. It is often said that the vermiform appendix, like the uvula, is useless. I do not believe, however, that there is anything in the human body but that has its uses. It is said to be an analogue; large in animals and in man a mere rudiment. It seems to me that it is a part of the intestines. It has an opening and has certain physiological uses. It does have inflammation quite frequently which may be due to the lodgment of dry fæcal matter within the appendix.

Dr. Chapman—Is it not on the increase at the present time? Do you not hear of more cases now than when you were a young man?

Dr. McNeil—I would like to ask Dr. Selfridge how many cases he has had, and how often has he operated for this condition?

Dr. Selfridge—I have had a great many cases. I have never operated for this condition as I have always been able to cure with my remedies.

Dr. Wilson—Were you always sure of your diagnosis?

Dr. Selfridge—Are we *sure* of anything? I think I can diagnose a case of appendicitis as well as I can anything.

Dr. McNeil—I have never had a case that I have diagnosed appendicitis. I think these cases will get well without an operation. I want to find out whether it is right to perform a laparotomy for every case supposed to be appendicitis.

Dr. Selfridge—You cannot tell that such a case is not appendicitis simply because you did not find it necessary to operate. A case of mine only last week. Patient with intense pain in the right iliac region, with swelling, distention, tympanitis, tenderness and flatness on percussion, fever, intense pain as if he had intussusception; he had always been constipated. I had the patient placed in position at an angle of about 45° and gave an enema, using plenty of water. My first prescription was *Plumbum-metallicum*$^{200}$, which I gave until the next day, when I found the inflammation to be more diffused; pain more paroxysmal; more tympanitis. *Colocynth* was indicated, which I gave in the 200th potency, and it cured. It controlled the inflammation. The patient was very sore for two or three days.

Dr. McNeil—What were the indications for *Plumbum?*

Dr. Selfridge—Constipation; severe pain, somewhat paroxysmal; and other symptoms calling for this remedy.

Dr. Chapman—The pain of *Plumbum* in bowel troubles is of a *drawing back to the spine* character.

Dr. Selfridge—The stool of *Plumbum* is like that of *Opium: pretty dark, lumpy balls.*

Dr. Chapman—In twenty-three years of practice I have not met a case that I have diagnosed appendicitis. I have had cases that looked like it, but I have always cured them with my remedies.

Dr. Selfridge—I had a case, not long ago, upon which I thought I would have to operate. There was present the marked swelling and every indication for an operation. The case got well after the administration of *Plumbum.*

Dr. Martin—A case came under my care ten days ago. A few days before that the patient ate very heartily of weddingcake, shrimp salad, and ice-cream ; drank considerable ice-cold beer, and as a result was seized with severe pain in the right iliac region, which diffused itself all over the abdomen. He was told by a physician to take six ounces of sweet oil, which he did without any result. The next day his family physician, Dr. V. C. Scott, was sent for, and free enemas were given which brought away large quantities of fæcal matter, and thoroughly cleaned the lower bowel. She also gave him *Colocynth*, which seemed to be indicated. She then called me in consultation. Upon examination I found the abdomen considerably distended and tympanitic, and extremely sensitive to touch, but the most sensitive part was in the right ilio-cæcal region ; the tongue was coated very brown ; temperature 102°. I suggested *Bryonia*, which was given. In the evening he was a little more comfortable. Dr. Bœricke was called in consultation the next afternoon, but the patient seemed a little better when he arrived. Dr. Bœricke agreed with the diagnosis, which was *appendicitis with complicating peritonitis.* The following morning the patient was much worse, and an operation was decided upon. I made a careful examination and found a tumor in the rectum. Dr. Bryant was called in the afternoon, but thought it too early to operate, so it was postponed until the next morning. The next day the patient was *very much worse*, and Drs. Bryant and Canney operated. As soon as the abdominal cavity was opened, the pus welled out in large quantities. The appendix was found sloughed off, and its distal end adherent to the intestines. Upon examination the appendix was found filled with dry, hardened fæcal matter, which was undoubtedly the cause of the inflammation. The patient was not able to withstand the shock of the operation and died early the next morning. Where there is general peritonitis with the appendicitis it is usually fatal.

Dr. McNeil—Is it normal for the appendix to contain fæcal matter ?

Dr. Selfridge—Yes. The appendix is open and there is nothing to keep the fæcal matter out. I believe there is peristaltic action in the appendix as there is in the cæcum.

Dr. McNeil—The structure would show whether there is any peristaltic action in it by the muscular fibre.

Dr. Chapman—Why is it necessary for it to have any function? It may be rudimentary like some of the muscles in the ear.

Dr. Selfridge—The muscles of the ear have their uses. I don't believe in the rudimentary idea, except, perhaps, in the fœtal circulation. I believe if you can see these cases of appendicitis in the beginning, you can cure them without operation. In the case that Dr. Martin recited, the enema should have been given first and not the oil. The cæcum should be washed out, and the indicated remedy given as well.

Dr. McNeil—The lodgment of the fæces causes the pain, and I think the indicated remedy would cure it.

Dr. Ledyard—I had a case of gastritis in which I used the indicated remedy. The patient had been suffering with gastritis and getting along very nicely, but had no operation of the bowels for a week. She told me about this, and I warned her against taking an enema even of water, as it would prevent the action of the indicated remedy. When I returned in the evening, I found that she had had an operation of the bowels during the day, and I supposed that it had come about naturally, but subsequently ascertained that she had used an enema. At 2 A. M. she awoke with intense pain, and the following night also, and that little enema threw her back at least three weeks. The enema removed the indications for the remedy, by suppressing some of the most important symptoms. I never succeeded in curing chronic constipation until I stopped all enemas. The enema ousts the remedy, so to speak, the more superficially acting agent gets in its work, and the remedy is thrown out. In a case of inflammation about a tooth, with considerable pain and swelling, a dentist extracted the tooth, and the patient had erysipelas which extended down to the collar bone. A dentist has no business to extract a tooth while there is any pain present, for in doing so he shuts up Nature's safety-valve. In a case of cauliflower cancer, the antitoxine of cancer was *injected into the cancer*, with the result that erysipelas flamed up with very painful symptoms which continued until death.

Dr. Chapman—Any indigestible matter remaining in the intestines I call a mechanical interference with the normal action of the intestines, and requires mechanical measures to remove it. I cannot believe that to remove it from the colon or appendix will interfere with the action of the remedy.

Dr. McNeil—I stand by Dr. Ledyard in regard to what he says in this matter. In the first place, when you remove the foreign substance from the bowels by mechanical means, you cover up some of your symptoms. To say that the indicated remedy will not remove the foreign body is an assumption. *The indicated remedy will remove it.* You cannot cure a case of constipation as long as you keep up the enemas. If you do Nature's work, she will stop doing it herself; and the consequence is that your indicated remedy will be useless. The homœopathic treatment will cure constipation. To say that it will not, shows a lack of faith in the homœopathic remedies.

Dr. Chapman—Here is a case. I was called to see a boy suffering most horribly; bowels would not move; intense tenesmus; it was evident that there was some foreign body in the bowels. With enemas and instruments I removed a lot of cinnamon bark and water-melon seeds. This was purely a surgical case. What would you do in such a case?

Dr. McNeil—Give my indicated remedy.

Dr. Martin—It takes a certain amount of time for the indicated remedy to act. When there is a stuffing up of the intestines causing inflammation, you must sometimes remove it before the indicated remedy will act. In regard to the cure of constipation; I have been very successful in these cases. I usually cure in a few weeks' time like this: If the patient goes five or six days without any movement of the bowels, and is suffering intensely, I give an enema perhaps two or three times, giving my indicated remedy all the while, and I know that it cures. By giving the enema and easing the suffering for the time being, the mental condition of the patient is relieved, which of course is quite a factor.

Dr. Ledyard—As far as the mental condition is concerned, the indicated remedy will remove *it* better and quicker than the enema can. In a case that came to my notice, a lady gave an enema to a little girl, which caused the fæces to *cut their way out.* I told her if she had waited it would have been better. The fæcal matter in such a condition will be so transformed by the indicated remedy that it will come away all right. Another case in which the patient did not have a stool for three weeks. He would always ask about having his bowels moved. I encouraged him by saying that he would be all right, and on the twenty-first day he had a natural stool; then a stool in seven days, then every two days, and finally every day.

Dr. Selfridge—I believe as much in the indicated remedy as any one; but I know that there is now and then a case in which the indicated remedy is not sufficient at the time. I had a case once of an infant to whom Castor-oil had been given, but without any effect. I then went prepared with a syringe, and gave an enema of soap and water, but it did no good. I then made an examination and found that the entire pelvis was filled with fæces; the rectum was completely plugged and nothing could pass. I was obliged to take a spoon-handle and scoop it out. It was as dry as a brick; all the fluids were absorbed. I dug it away until I got through the dry part, and then it came out with a gush all over my hand. I do not believe that any indicated remedy would have relieved that child.

Dr. McNeil—I believe that the indicated remedy would have relieved the child. I will not let my patients use enemas.

Dr. Selfridge—In such a case no medicine on God's earth would cure it. The fæces in this case formed a surgical plug, and had to be removed by surgical measures.

Dr. Martin—What about stone in the bladder? We know that the indicated remedy is often of no use until the stone is removed by surgical means.

Dr. McNeil—That is surgical.

Dr. Ledyard—It is the same in treating displacements of the uterus. You cannot cure a prolapsed uterus with a pessary.

Dr. Martin—In some cases a pessary is absolutely indicated.

Dr. Selfridge—We must all use judgment in our cases.

Dr. Chapman—I believe that in some cases the indicated remedy is surgery.

Dr. McNeil—I will relate the case of a woman after abortion who had been flowing for a long time, and waited too long for me. When I saw her she said, "If I move the very joints of my fingers pain." I gave her *Bryonia*. If I could not find my remedy I would use tampons, but there are remedies for these cases.

Dr. Selfridge—There are cases in which the indicated remedy will not act.

Dr. McNeil—In Hering's *Guiding Symptoms*, there are remedies mentioned for these bowel conditions; for instance, *Silicea*, *Sepia*, *Aloes*, the indication for them being, *fæces cannot pass without being removed mechanically*. These are from clinical provings; cases in which that condition existed, and these remedies were given and cured.

Dr. Selfridge—In all those cases it was a paralysis of the rectum, and not a plug that caused the trouble. In such a case the indicated remedy would remove the trouble.

Dr. Martin—I had a case where the fæces would act as a plug in the rectum, and Aloes stopped the tendency to this formation.

Dr. Ledyard—If the indicated remedy will remove a large tumor, why cannot it remove the fæces?

Dr. Selfridge—It cannot remove the fæces when it acts as a plug in the rectum. The secretions have all been absorbed, and it needs moisture to soften up the hardened mass.

Dr. Underwood—If the indicated remedy is set to work, will it not increase the secretion, thus causing the fæces to come out naturally?

Dr. Selfridge—It might if the stool is not so large that it *cannot* come out.

Dr. Chapman—How about my water-melon seeds?

Dr. Underwood—I think the increased secretion would break up the mass so that they would pass out, too.

Dr. Martin—In the case you related, Dr. Selfridge, how long had the fæcal plug been in that infant's rectum?

Dr. Selfridge—They had been giving it Castor-oil for weeks.

Dr. Martin—Castor-oil will stimulate the intestines, and if moisture was all that was needed there ought to have been enough; hence it proves that it had to be removed mechanically.

Dr. McNeil—I wish to state that the warts that have been on the top of my head for five or six months have been removed by one dose of *Sepia*$^{200}$. There were twenty-five of them of good size, with groups of smaller ones.

The Secretary then read a letter from Mr. E. R. Lilienthal, thanking the members of the Club for the resolutions passed regarding the death of his brother, Dr. James E. Lilienthal. The President ordered that the letter be placed on file.

Upon motion of Dr. Martin, seconded by Dr. Wilson, the meeting then adjourned to meet at the office of Dr. A. McNeil, 784 Van Ness Avenue, San Francisco, the third Friday in November.

